# Closed-Loop Neuromorphic Benchmarks

**DOI:** 10.3389/fnins.2015.00464

**Published:** 2015-12-15

**Authors:** Terrence C. Stewart, Travis DeWolf, Ashley Kleinhans, Chris Eliasmith

**Affiliations:** ^1^Centre for Theoretical Neuroscience, University of WaterlooWaterloo, ON, Canada; ^2^Mobile Intelligent Autonomous Systems group, Council for Scientific and Industrial ResearchPretoria, South Africa

**Keywords:** neuromorphic hardware, benchmarking, minimal simulation, adaptive control, neural networks

## Abstract

Evaluating the effectiveness and performance of neuromorphic hardware is difficult. It is even more difficult when the task of interest is a closed-loop task; that is, a task where the output from the neuromorphic hardware affects some environment, which then in turn affects the hardware's future input. However, closed-loop situations are one of the primary potential uses of neuromorphic hardware. To address this, we present a methodology for generating closed-loop benchmarks that makes use of a hybrid of real physical embodiment and a type of “minimal” simulation. Minimal simulation has been shown to lead to robust real-world performance, while still maintaining the practical advantages of simulation, such as making it easy for the same benchmark to be used by many researchers. This method is flexible enough to allow researchers to explicitly modify the benchmarks to identify specific task domains where particular hardware excels. To demonstrate the method, we present a set of novel benchmarks that focus on motor control for an arbitrary system with unknown external forces. Using these benchmarks, we show that an error-driven learning rule can consistently improve motor control performance across a randomly generated family of closed-loop simulations, even when there are up to 15 interacting joints to be controlled.

## 1. Introduction

Neuromorphic hardware holds great promise for a wide variety of applications. The combination of massively parallel computation and low power consumption means that there is the potential to have complex algorithms running in embedded processing situations, without being a significant drain on available energy. A crucial challenge is to identify what sort of always-on or interactive functionality can best exploit these devices.

To evaluate applications of neuromorphic hardware, we need benchmark tasks. These tasks must allow us to compare across different instances of neuromorphic hardware (and potentially across different algorithms implemented in that hardware). Good benchmarks will allow us to quantitatively compare systems, letting researchers both measure the progress in the field, and also directly compare competing approaches.

In this paper, we focus on the development of *closed-loop* benchmarks. These are dynamic tasks where the output of the neuromorphic hardware *influences its own future input* through some environment. This is in contrast to standard categorization or pattern identification tasks, where the input is some fixed sequence and the hardware must produce the correct output for each input (or input pattern).

We believe closed-loop benchmarks should be of particular interest to neuromorphic research, given that the most compelling applications of neuromorphic hardware are likely to be in this domain of embedded and interactive control of robotic or other physical systems. However, the closed loop itself raises a number of issues that complicate the development of such benchmarks. Rather than simply providing a data file of inputs and desired outputs, the benchmark must either specify a full physical system to be controlled, or it must provide software for a simulation of that system. As we discuss below, both approaches are problematic. Describing a method for overcoming these shortcomings is the primary goal of this paper.

## 2. Closed-loop benchmarks

A closed-loop benchmark task is one where the system we are studying has a two-way interaction with some sort of environment. That is, the outputs from the neuromorphic hardware are sent to the environment where they cause an effect, the results of which change the subsequent input. For example, the outputs might control the movement of a robot, which in turn affects the sensory data received by the robot.

### 2.1. Simulation vs. physical instantiation

To define a closed-loop benchmark, we need to be explicit about the interaction with the environment. If a robot is to be controlled, we need to specify all of the details of that robot. What motors does it have? How are they configured? How strong are they? What sensors are there? Where are they placed? How accurate are they? However, even if these questions are answered, there is a fundamental problem in that *other researchers need access to that exact robot*. If a benchmark is to be widely used, other researchers developing their own neuromorphic hardware should be able to do their own testing on the same benchmark system.

Furthermore, using a physical robot imposes significant practical difficulties when performing extensive benchmark testing. When testing, we often want to run the same task over and over again, both for robustness and to see the effects of varying parameters. With a physical robot, this means manually setting up the task, letting the test run, gathering the resulting data, and then resetting the robot back to the initial state. Consequently, issues like battery life become problematic, and not just because there is a limited amount of time available for testing. As the battery level changes, the performance of the robot itself can also change. Futhermore, for any rigorous testing of the benchmark, we will want to examine situations where the system fails. This means that some of the testing will involve parameter settings that lead to poor behavior, which might have the undesirable result of causing physical damage.

However, *not* using a real physical embodiment for testing is also problematic. First and foremost, without an actual real-world task, why should we have any confidence that the performance on the benchmark is reflective of the actual usefulness of the neuromorphic hardware? It is widely known that simulations of robots (or other physical systems) are often *much* easier to control and better-behaved than the real thing (see Jakobi et al., 1995; Koos et al., 2013). The field of robotics is filled with algorithms that work well “in theory,” but fail when run on actual hardware. We do not want a benchmark that falls into this trap, giving high scores to hardware that does not turn out to function well when deployed in real situations.

A variety of robotics simulators, such as Webots and Gazebo, already exist and are intended for evaluating robotics performance. These are extremely useful, but have two important limitations. First, they are generally meant to evaluate *one particular* robot body, and it is difficult to, for example, automatically generate a large number of different physical bodies to evaluate over. This means that such a system is good for evaluating a particular control system for a particular robot body, but is not suitable for the more general question of how well the control system will work over a large space of different robot bodies. Second, these simulators tend to run slower than real-time. Typically, when a simulation is too simple to reflect reality, more details are added to the simulation itself. Incredibly finely detailed simulations can be created, filling in all of the details needed. However, accurate modeling of physical systems can very quickly become *impractical to run in real time*. This is a fundamental problem, in that neuromorphic hardware is often tied to real-time interactions, and there can be no way to slow down the hardware to match the simulated environment. This means that even if we spent the considerable amount of research effort needed to define a simulated environment for a closed-loop benchmark, running that simulation fast enough to interact with the desired hardware would require significant computing resources. Indeed, one of the major efforts in the Neurorobotics section of the Human Brain Project is to develop exactly this sort of computing infrastructure (Hinkel et al., 2015), with a dedicated supercomputer to run the physics simulations. Until this hardware is publically available (and until software is available to create a variety of physical robot models), this approach is problematic for other researchers.

We are thus left with a situation where any benchmark we might define for a closed-loop task will be impractical for different researchers to run (if it is physically embodied), inapplicable to real-world situations (if it is a simulation that is simple enough to run in real-time), or impossible to connect to some neuromorphic hardware (if it is a simulation that runs slower than real-time). We thus need a new approach to provide a sharable real-time simulation that is robust enough that neuromorphic hardware that learns to deal with the simulation might also be able to deal with reality.

### 2.2. Minimal simulation

The above considerations could be taken as an argument that even though using real-world physical hardware for benchmarking is problematic, it is still better than using simplistic simulations which may not generalize to real tasks. However, we do not think this is the case. Instead, we believe neuromorphic benchmarking can effectively exploit an approach known as *Minimal Simulation* (Jakobi, 1997).

This approach was first suggested in the context of evolutionary robotics. Notably, the problem faced by closed-loop neuromorphic benchmarking is remarkably similar to that faced earlier by these researchers. In evolutionary robotics, the goal is to use genetic algorithms to *evolve* systems that can control robots to perform various tasks. These tasks can be as simple as navigation and obstacle avoidance, but have also included more difficult tasks such as walking, collecting objects, and visual tracking (Nolfi and Floreano, 2000).

However, performing evolution on real physical robots is problematic for the same reasons that benchmarks on physical robots are problematic. The robots must be reset to the same state each time; they often involve behavior that can physically damage the robots; and they take a very long time to run. For this reason, attempts were made to evolve algorithms using simulated robots. However, the general finding was that algorithms that worked on the simulated robots would not work when run on the real physical robots. If the simulations were improved, adding complex physical detail, then it was possible to generalize to real behavior; unfortunately, such complex simulations would run slower than real-time (see Husbands and Harvey, 1992; Husbands et al., 1993).

To address this problem, Jakobi (1997) proposed the creation of “minimal” simulations. These are simulations where there is variability *within the simulation itself*. In other words, we make *poor* simulations, but ensure that the way in which they are poor is itself variable. We are then in a position to ensure that the controllers work across that whole range of variability. “Instead of trying to eliminate the differences between simulation and reality, they are acknowledged, and mechanisms are put in place to prevent evolving controllers from relying on them (Jakobi, 1998, p. 48).”

With this approach, it became possible to build minimal simulations that would run faster than real-time and yet also be complex enough that if a system could successfully control the simulation, it was also likely to successfully control a real robot. To achieve this kind of transfer, the simulations were made to be unreliable in almost every respect. For example, for a simulation of a simple motor it would still be the case that if power is applied it would generally try to spin, but the exact amount of torque, the amount of sensory noise, the amount of time needed, the amount of static and dynamic friction, and so on would all be randomly chosen. A successful controller would have to deal with this wide range of variability, and if it could handle that variability then there would be reason to believe it could also handle the real physical system.

It is worth noting that a minimal simulation only has to be a good simulation *for successful behavior*. That is, “if we are evolving corridor following behavior, the dynamics of the simulation might differ wildly from those of reality if the controller hits a wall or goes round in circles, but this does not matter, since the controllers we are interested in transferring across the reality gap will neither hit walls nor go round in circles (Jakobi, 1998, p. 41).” If the controller is poor, we do not need the simulation to be at all accurate in exactly *how* that poor behavior is manifest. We do not need an exact detailed physics model of the collision between a robot and a wall, or a detailed model of what happens to a robot arm when it starts oscillating wildly due to a poor control signal. All we need is for the simulation to be just good enough to indicate that things have gone wrong, and thus give a low score to that controller. This means that, for example, in a minimal simulation of an eight-legged walking robot, it is not necessary to have a physics simulation that correctly models what happens when two legs collide with each other. Rather, if legs collide with each other, that is an indication that the walking behavior is very poor. As long as that result is indicated we can greatly simplify the simulation by not including all the details necessary to model these complex physical interactions. This approach was successfully used to develop models of multi-legged walking robots (Jakobi, 1998; Meyer et al., 2003) and vision-based tracking of a moving object (Nolfi and Floreano, 2000).

### 2.3. Minimal simulation for benchmarking

Although minimal simulation has not previously been used outside the domain of evolutionary robotics, we propose using minimal simulation for neuromorphic benchmarks. We would argue that one important use of a benchmark is *generalization*. That is, by knowing how well particular hardware performs on a benchmark, you can reasonably infer how well that hardware will perform in other situations. For example, if an image recognition algorithm performs well on the MNIST hand-written digit recognition benchmark, this suggests that it may also perform well on a different recognition task. Of course, this inference will fail if that algorithm has been specifically over-fit to that situation. For that reason, it is useful to have benchmarks that cover a wide range of variations on the task. If the hardware performs well across that variability, then it is more likely to also work in sufficiently similar new situations.

To achieve this kind of transfer, we need software simulations of the environment for the task. These simulations must be fast enough to run in real time (so that they can be controlled by real neuromorphic hardware), and they must be extremely variable, to encourage robustness of the methods being benchmarked. Each time the simulation is run, different parameters will be chosen to give significant variability (so one run might have a large degree of sensor noise while the next run has none at all; one run might have more delay in the motor response and another might have less power available). Being successful at the benchmark means being successful across all this variability.

The result is a benchmark that can be used by any researcher. The fact that it is a simulation means that source code can be shared, and that no specialized hardware is needed. Furthermore, the variability in the simulation itself can be controlled, and this can help give a rich characterization of the benchmarked hardware. For example, some hardware might only work with small amounts of sensor noise, while other hardware might be most effective when there is significant delay in the motor response. This flexibility in parameters in the benchmark allows researchers to explicitly characterize those particular situations where their hardware excels.

### 2.4. Cost-effective robotics

The minimal simulation described above forms the core of our method for generating benchmarks. The purpose of these benchmarks is that is that they should do a reasonable job of generalizing to real-world physical tasks. Consequently, it is important to supplement simulation benchmarks with at least one easy-to-construct physical analog. This physical version would be one particular instance of the type of situation the benchmarks are meant to cover. For that reason, the physical task is much more restrictive in terms of what general conclusions can be drawn from how well different hardware performs in that situation. Rather, the purpose is to give an explicit double-check that hardware that performs well on the simulation benchmarks also perform well in a physical environment.

To keep the physical aspect simple, we recommend cheap, cost-effective, widely-available components. This allows a greater chance for other researchers to have access to the same (or similar) hardware. For the particular example benchmark described in the next section, we use the Lego Mindstorms EV3 kit, a simple robotics platform available at most toy stores.

It is important to note that there is a theoretical advantage to using simple robotics hardware for benchmarking, in addition to the practical advantages. In particular, we *do not want benchmarks that rely on high-speed, high-accuracy devices*. The purpose of benchmarks is not to indicate how well this neuromorphic hardware works to control this particular robot in this task. Rather, the purpose of a benchmark is to characterize how well some specific neuromorphic hardware works on a task *in general*. The variability introduced in the minimal simulation means that the hardware should be able to function across a wide variety of physical embodiments, and so if we are to choose one particular physical embodiment to test in the real world, then we should choose one that is not high-precision. For this reason, we believe using Lego robots is actually more informative for benchmarking than expensive high-precision robots^1^.

## 3. Example: adaptive motor control

To demonstrate this approach to creating closed-loop neuromorphic benchmarks, we now consider a basic control task. Suppose we have a system with a number of joints with positions *q* = [*q*_1_, *q*_2_, …, *q*_*n*_] and we want to send a control signal *u* = [*u*_1_, *u*_2_, …, *u*_*n*_] to the motors at each joint such that the joints move to a particular desired position *q*_*d*_ = [*q*_*d*, 1_, *q*_*d*, 2_, …, *q*_*d, n*_]. The only output from the controller is the signal *u* and the inputs are the current position of each motor *q* and the desired positions *q*_*d*_.

The simplest controller for such a situation is a P (proportional) controller, where *u* = *K*_*p*_(*q*_*d*_ − *q*). This is often supplemented with a D (derivative) term, which helps to slow the system down as it approaches the desired position, thus avoiding overshoot and oscillation. This combination of terms leads to the standard PD controller $u=K_{p}\left(q_{d}-q\right)+K_{d}\left(\dot{q_{d}}-\dot{q}\right)$. Both *K*_*p*_ and *K*_*d*_ are constants that can be tuned to particular situations.

**Figure 1:**
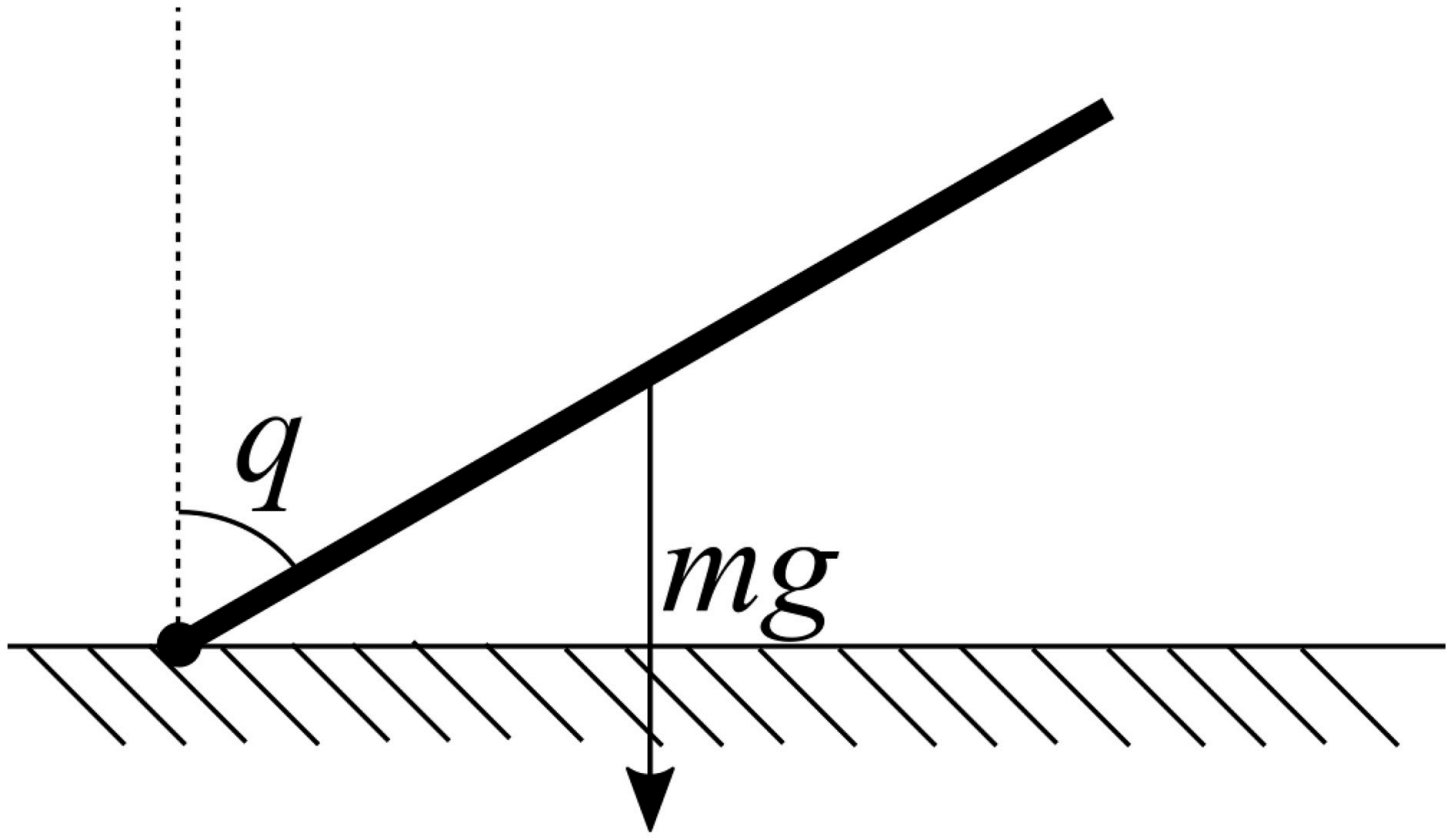
**A simple example of a physical embodiment for control**. In order to hold the joint *q* at a desired angle *q*_*d*_, a force must be applied to counteract the pull of gravity. The magnitude of this force is a function of *q* which can be learned.

However, this controller has difficulty in the presence of significant external forces. For example, consider a single motor controlling the angle of a single arm. If the arm is held out to the side, gravity acting on the mass of the arm itself will pull the arm downward. Thus, to hold the arm still at a particular *q*_*d*_ will require the controller to apply a force to counteract gravity. Since the PD controller always produces an output *u* = 0 when *q* = *q*_*d*_, it cannot compensate for gravity, and so the arm will stay stationary at some angle below the desired angle (Figure 1).

The standard solution to this steady-state error is to add an I (integral) term (*K*_*i*_ ∫ (*q*_*d*_ − *q*)*dt*) to the controller, making it a PID controller. As the difference between where it is (*q*) and where we want it to be (*q*_*d*_) accumulates over time, the *K*_*i*_ term will gradually increase the extra controlled force *u* that is being applied until it is large enough to counteract the external force of gravity (or whatever other external forces are present). However, this approach has great difficulty when *q*_*d*_ changes, since the external force due to gravity changes depending on the position of the arm *q*. The controller ends up having to “relearn” the correct amount of extra force needed every time *q*_*d*_ changes.

In some robotics applications, this problem is solved by mathematically analyzing the geometry and mass of the system to compute exactly how much extra force is needed. In this particular case, the answer is straight-forward, in that the extra torque due to gravity is $\tau =mg\frac{l}{2}sin\left(q\right)$, where *m* is the mass of the arm, *l* is the length, and *g* is 9.8 m/s^2^. If the force applied by the motor is linear in *u*, then we could simply compute this value and add it to our controller's output. However, this assumes a perfectly even distribution of weight in the arm, ignores momentum, friction, and other forces, and gets much more complex as more joints are added. Furthermore, if this initial computation is slightly off, or if details of the system change, there is no way to adjust this compensation.

Fortunately, there is an adaptive solution to this problem, and it is one that fits well with neuromorphic hardware. Slotine and Li (1987) show that if you express the influence of the external forces as τ = *Y*(*q*)ω [where *Y*(*q*) is a fixed set of functions of *q*, such as *sin*(*q*), and ω is a vector of scalar weights, one for each function in *Y*], then you can learn to compensate for these external forces by using the learning rule Δω = α*Y*(*q*)*u*, where *u* is the basic PD control signal.

Importantly, as pointed out by Sanner and Slotine (1992) and Lewis (1996), rather than making explicit assumptions about the exact functions that should be in *Y*(*q*), we can use a neural network approach where each neuron is a different function of *q*. As long as there is enough hetereogenetity (i.e., as long as the neural activity forms a basis space that is capable of approximating the external forces), then the learning rule will continue to work. This approach has been extended to biologically plausible neurons and been used in both the Recurrent Error-driven Adaptive Control Hierarchy (REACH) model of human motor control (DeWolf, 2014) and quadcopter control (Komer, 2015).

These considerations suggest that there is a neuromorphic-friendly family of algorithms to address the general problem of controlling a wide variety of physical systems. Identifying those algorithms will allow us to benchmark their performance across example tasks and physical configurations. To implement these algorithms, the input to the neuromorphic hardware is *q*, the system state. This input is fed to each neuron such that each neuron produces some output activity that is based on this input. Since *q* will be multi-dimensional (if there is more than one joint), we may give each neuron a random weighting of each *q*-value (*J*_*i*_ = *e*_*i*_ · *q* + *b*_*i*_, where *J*_*i*_ is the input to neuron *i*, and *e*_*i*_ is a randomly chosen vector^2^, and *b*_*i*_ is a randomly chosen bias term). Given this input, the neurons will produce some output *A*. We now form a weighted sum of these outputs *Ad*, where *d* is a matrix (number of neurons by number of elements in *q*) that is initially all zeros.

To use this controller, we add its output to that of the standard PD controller. That is, the standard controller has $u=K_{p}\left(q_{d}-q\right)+K_{d}\left(\dot{q_{d}}-\dot{q}\right)$, and so our actual output to the motor is *u* + *Ad*. We then apply a learning rule on *d* such that Δ*d* = α*A* × *u*. Here, α is a learning rate and the cross product is used so that we are applying the learning rule on all the joints simultaneously.

Notice that we can think of this system as a three-layer neural network, where the input and output layers are linear. The first layer is *q*, the input state, one value for each joint. The “hidden” layer is the neurons producing activities *A*, the activity of a large number of neurons. The output layer again has one value per joint, and is the extra added signal to apply to the motors, *Ad*. Given that this is a canonical example of the use of neural networks, we expect that the majority of neuromorphic hardware is flexible enough to implement this model. Importantly, it functions well with spiking neuron models as well as non-spiking ones. For spiking neurons, we consider *A* to be the instantaneous measure of the output of a neuron (i.e., whether or not it is currently outputting a spike), filtered through a low-pass filter. Further discussion of this sort of learning rule and comparison to biological spiking neurons can be found in Bekolay et al. (2013). This type of neural modeling forms the foundation of Eliasmith and Anderson (2003)'s Neural Engineering Framework, which has shown that spiking and non-spiking neurons can be used in this manner to implement a wide variety of computations (e.g., Stewart and Eliasmith, 2014).

It should be noted that, while this algorithm fits well into neuromorphic hardware, other hardware might be better (in terms of accuracy, energy efficiency, cost, or even development time). Answering this sort of question is exactly why we need to use a benchmark that can compare multiple different hardware implementations of this algorithm. Furthermore, since some hardware may be better in different situations, we need a benchmark that has flexible parameters, rather than one that is based on a single particular physical system.

### 3.1. Online and offline learning

The rule for modifying the weights *d* described here is of a very common form, as the weight update from a neuron is proportional to the activity of that neuron and an external error signal. This makes it an instance of the ubiquitous delta rule. Thus, neuromorphic hardware that has built-in learning will often be able to natively support this rule. However, some neuromorphic hardware does not intrinsically have the ability to update connection weights in this manner.

In that case, there are at least two possible ways to implement this algorithm. First, the multiplication by *d* can be done on the output from the neuromorphic hardware. Any closed-loop neuromorphic system will have some method that takes the neural output from the hardware and sends it to the motors (or to the simulation of the motors). Instead of sending the result of *Ad*, the hardware could send *A* (the activity of all the neurons), and the interface to the motor can be responsible for doing the multiplication by *d* and updating *d* according to the learning rule.

Alternatively, it may be possible to use offline learning. That is, rather than updating the weights *d* during the simulation, we record *A* and *u*, and after a period of time stop the controller, compute the sum of the changes to *d*, load the new value of *d* onto the neuromorphic hardware, and start the controller again.

Given this variety of options for implementing adaptive algorithms of this type, we believe it should be possible to benchmark most neuromorphic systems on adaptive control tasks in this manner.

### 3.2. Minimal simulation for adaptive control

Now that we have defined the task domain, we can use the principles of minimal simulation to construct a flexible and variable simulated environment for testing adaptive control. In this case, we would like to develop a bare-bones simulation of the system being controlled, with significant variability. If the neuromorphic controller works well across this variability, then it is likely to work well outside of simulation as well.

The basic system variable is a vector of joint angles *q*. Each joint has a velocity *v*. The force applied by each motor is related to the signal *u* sent to the motors, but will generally have some maximum value *T*, so we use *tanh*(*u*)*T* to determine the force as a function of the control signal. To account for friction, we scale the velocity by some factor *F* every time step. This results in the simplistic simulation described by:

(1) Δv =−vF+tanh(u)T

(2) Δq =v

In addition, we add an external perturbing force. In a real system, this could be the effects of gravity given the current configuration of the motors, or of other unexpected influences. Rather than choosing one particular fixed external force for our benchmark, we *randomly generate* this force each time the benchmark is run. This ensures that the benchmark covers a wide range of possible external forces and motor configurations, rather than just one particular situation.

Specifically, to generate this force, we start with a small set of smooth functions *f* which are often found in dynamics equations (*x*, *x*^2^, *sin*(*x*)). We then generate an external force of *K*_*f*_(ζ · *f*(β · *q* + γ) + η) where ζ, β, γ, and η are all random vectors and *K*_*f*_ is a scaling factor to control how strong the external force is. The result is added to Equation (1). For example, if *q* is 4-dimensional (i.e., if there are four joints being controlled) and if there are three smooth functions in *f*, then β, γ, and η are all vectors of length 4 and ζ is a 4 × 12 matrix. To introduce significant variability, all of these values are randomly chosen from the normal distribution *N*(0, 1).

To complete the simulation, we introduce additional sources of variability: random noise, delay, and filtering to both the input and the output of the system. For noise, we add *N*(0, σ_*u*_) to the control signal *u* and *N*(0, σ_*q*_) to the *q*-value reported back to the controller. We also use a low-pass filter to smooth both values (with time constants τ_*u*_ and τ_*q*_) after this noise is added, giving a damping effect. Finally, both *q* and *u* are delayed by an amount of time *t*_*q*_ and *t*_*u*_ to reflect communication delays that are common in physical systems.

The resulting simulation is not meant to be an accurate portrayal of a particular physical embodiment. Rather, this simulation is meant to be extremely fast to simulate, and it is meant to be similarly difficult to control as a real system. In other words, if a controller manages to be able to control the various randomly created minimal simulations of embodiment that are generated with this approach, then we have reason to believe that it will also be successful at controlling real embodiments. With this minimal simulation and modern computers, we can run real-time simulations of systems with dozens of joints that have highly complex interactions between them. Consequently, we can effectively benchmark how well an adaptive controller deals with these situations.

### 3.3. Calibrating the minimal simulation via cost-effective robotics

It is important to ensure that the minimal simulation defined in the previous section is representative of the sorts of real-world situations in which we want to use these same controllers. Importantly, this physical instantiation does not have to exactly match any particular parameter setting of the minimal simulation. Rather, we want a physical system that shares basic functional similarities to the minimal simulation defined previously.

For example, we want the inputs to the system to act like *u*, in that a positive number will increase some velocity *v* which will in turn increase some sensor value *q*. We want there to be some sort of external applied force that affects *q*, and we want that external force itself to be a function of *q*. We want there to be communication delays and noise in the sensor and motor systems, and we want all of these effects to be somewhere within the ranges covered by the minimal simulation. While implementing this kind of hardware analog cannot guarantee that neuromorphic hardware that is successful in simulation will be successful in every similar real-world task, it does provide an existence proof that there is at least one real-world task where the hardware performs similarly to how it performs in simulation.

**Figure 2:**
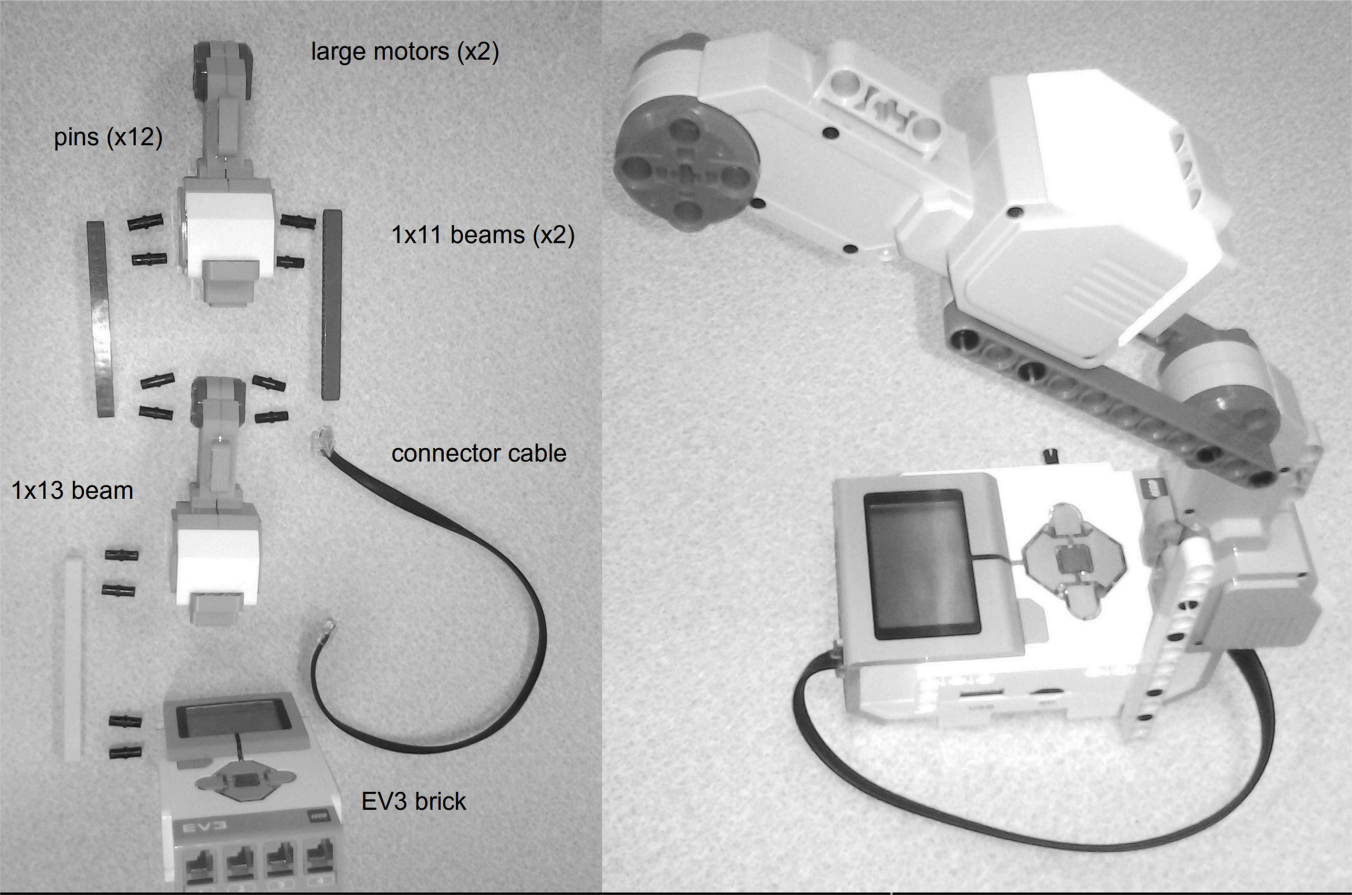
**A simple physical robot embodiment for calibrating the minimal simulation**. All components come with the Lego Mindstorms EV3 kit, and are shown on the left (2 large motors; 2 1x11 beams; 1 1x13 beam; 12 pins; 1 EV3 brick; 1 connector cable). To rotate the central motor to the desired position *q*, enough force must be added to *u* to counteract the weight of the second unused motor.

For our specific demonstration, we describe an easy-to-build system that can be usefully controlled by this adaptive method. In particular, we use the Lego Mindstorms EV3 robot kit, organized as shown in Figure 2. It consists of a single motor, mounted such that the full weight of a second (unused) motor applies a significant force on the arm itself. Multiple motors can be added, and other configurations can be considered and should also be suitable for benchmarking, but here we consider only this basic case.

To interface to the physical hardware, we installed the ev3dev operating system (http://ev3dev.org), a Debian-based Linux system specifically developed for the EV3. We then installed and ran the ev3_link program from ev3dev-c (https://github.com/in4lio/ev3dev-c). This allows the EV3 to listen for UDP commands that tell it to set motor values and read sensor values. Communication with a PC was over a USB link (although the system also supports WiFi communication). With constant communication, the system is able to adjust the power sent to the motors *u* and give position feedback *q* from those motors at a rate of around 200 Hz.

**Figure 3:**
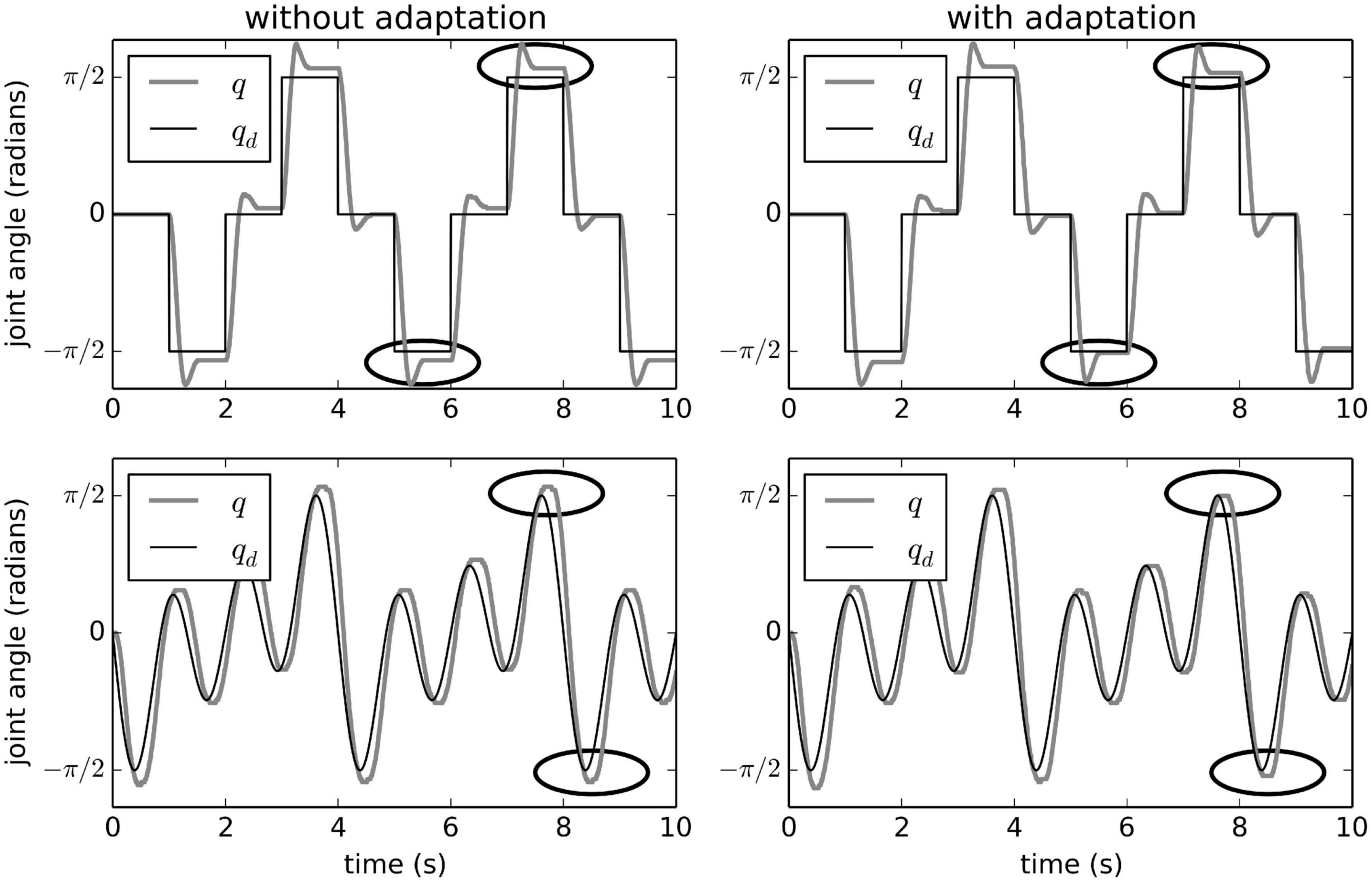
**Adaptive control of the EV3 lego robot used for calibrating the minimal simulation**. The effects of adaptation over two different desired trajectories are shown. Without adaptation, the joints *q* do not reach the desired *q*_*d*_ when *q*_*d*_ is large (which is when the external force is largest). With adaptation, *q* is closer to *q*_*d*_ after about 5 s, showing that the system has quickly learned to compensate. Points in time where the improvement is clearest are circled.

Figure 3 shows the effects of adaptive control on this physical system. Without adaptation (i.e., with a simple PD controller), there system state *q* (the joint angle) overshoots the desired *q*_*d*_. This overshoot is largest when *q* is large. This is because the external force applied to the joint due to gravity is proportional to *sin*(*q*). The *q*-value also overshoots and comes back part-way, due to physical momentum.

**Figure 4:**
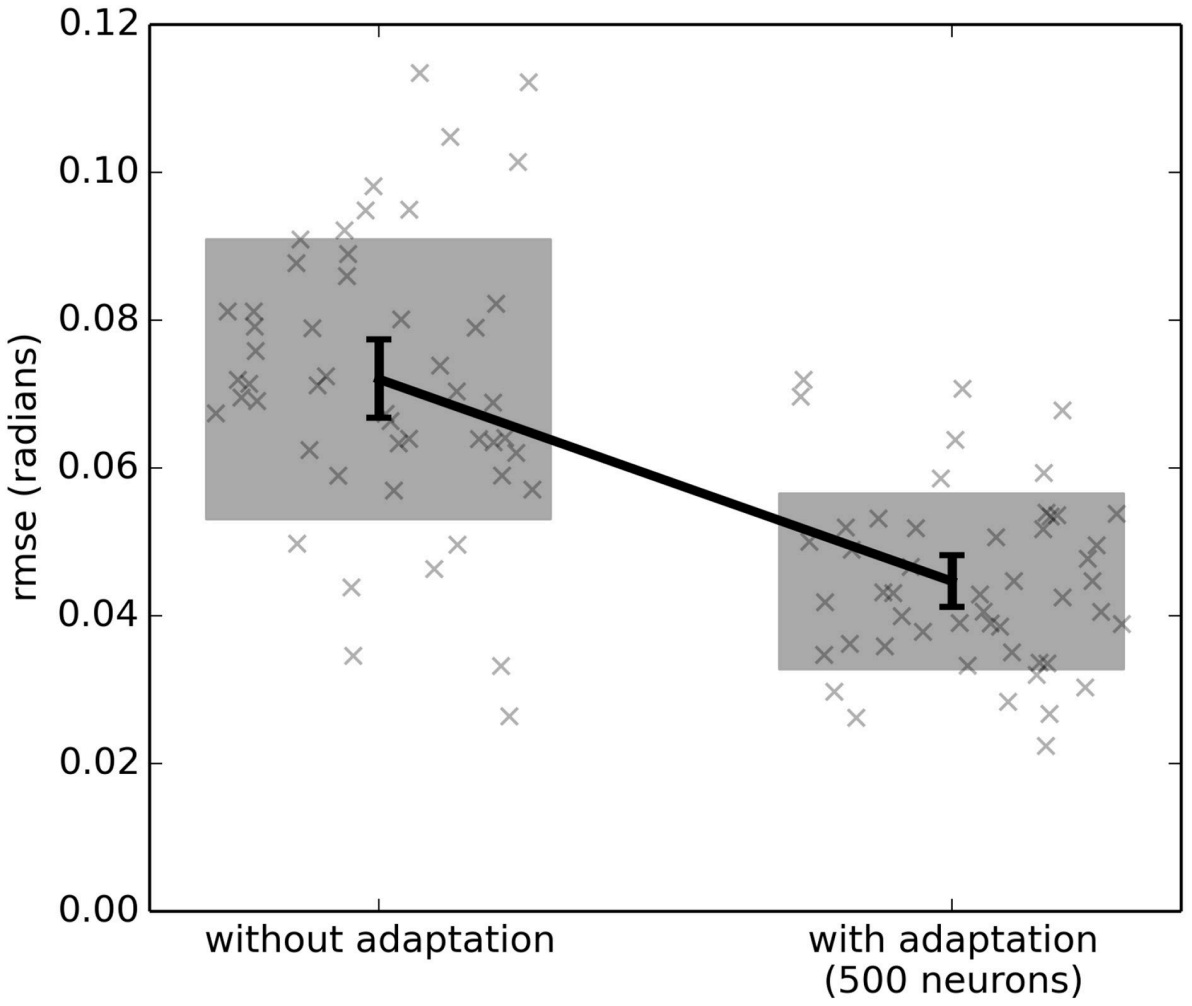
**The effect of adaptive control on a single-joint lego robot (Figure 2)**. Each run uses a randomly generated desired trajectory *q*_*d*_(*t*) over 20 s, and root-mean-squared-error is computed over the last 10 s only. The adaptive algorithm provides a significant improvement (*p* < 0.05; two-tailed *t*-test; 50 samples). Scatterplots show individual runs (with random jitter on the x-axis to avoid overlap), the shaded area is the mean plus or minus the standard deviation, and the 95% confidence interval of the mean is shown.

However, with adaptation (the right-hand side of Figure 3), the system learns to counteract this extra force due to gravity. After the first 5 s, the system is able to bring *q* much closer to the desired *q*_*d*_. Figure 4 shows the average improvement over 50 experimental runs with different randomly-generated desired target paths *q*_*d*_(*t*). Adaptation provides a clear improvement.

Now that we have this physical example of the task out minimal simulation benchmark is meant to cover, we can use it to calibrate the parameters of the simulation. For example, to characterize the communication delay between the computing hardware and the EV3 robot, we simply measure the number of times per second we can send a motor command *u* and read the position of the motor *q* per second. This works because the ev3_link software is entirely synchronous and only responds with motor positions when it processes a command to do so. This rate of communication averaged 154–156 Hz (95% bootstrap confidence interval over 100 trials) with a standard deviation of 3.3–4.7 Hz (95% bootstrap C.I.). This indicates a round-trip delay on the order of 0.006 s. Given this, we set the delays in the simulation to be uniformly chosen between 0 and 0.01, so that the minimal simulation covers delay conditions even worse than those seen in the EV3 robot.

For sensor noise, we note that the EV3 rotation encoders for the motors (the devices that measure *q*) have a resolution of 0.0175 radians (1 degree). This is a very different sort of noise than the Gaussian noise used in the simulation, so we set the simulation noise σ_*q*_ to be much larger (uniformly distributed between 0 and 0.1). Similarly, the motor resolution is 0.01, as it accepts integer values up to 100, so we set the motor noise σ_*u*_ to be uniform between 0 and 0.1.

Finally, we can use the physical system to calibrate the relationship between *T* (the maximum torque applied by the motor) and *K*_*f*_ (the scaling factor of the external force). After all, we do not want external forces that are so strong that the system does not have enough strength to counteract them. To measure this on the physical robot, we applied a standard PID controller with a target *q*_*d*_ of π∕2 (the position at which maximum torque must be applied to counteract gravity). After giving the system 5 s to stabilize, we recorded the required motor command sent to the robot (from −1 to +1). On average, this was 0.11–0.16 (95% bootstrap confidence interval over 50 trials), with a standard deviation of 0.07–0.12 (95% bootstrap C.I.), and a maximum value of 0.36. Considering this a worst-case scenario, if we arbitrarily fix *K*_*f*_ to 1 and randomly generate external forces given the process described above, then 95% of the time we get values between −3.75 and +3.75. Since we want the motors to be strong enough to compensate for forces in that range, we set *T* to 10 (≈3.75/0.36).

## 4. Benchmark analysis

To run a benchmark using the proposed minimal simulation approach, there are four main steps: (1) identify the neuromorphic hardware to be tested; (2) construct the minimal simulation; (3) determine a metric (e.g., root-mean-squared error; rmse) to record; (4) specify distributions for any parameters in the simulation. We then perform multiple runs of the simulation, each time choosing different values from the parameter distributions. For each run, we reset the hardware to its initial state, so there is no learning from one run to the next. This means our metric indicates how well the system will perform on a single environment, rather than attempting to use the same learned parameters across different environments. We can then plot how the metric varies as a function of a particular parameter of interest, or how it compares across different hardware for a given set of parameter distributions.

**Figure 5:**
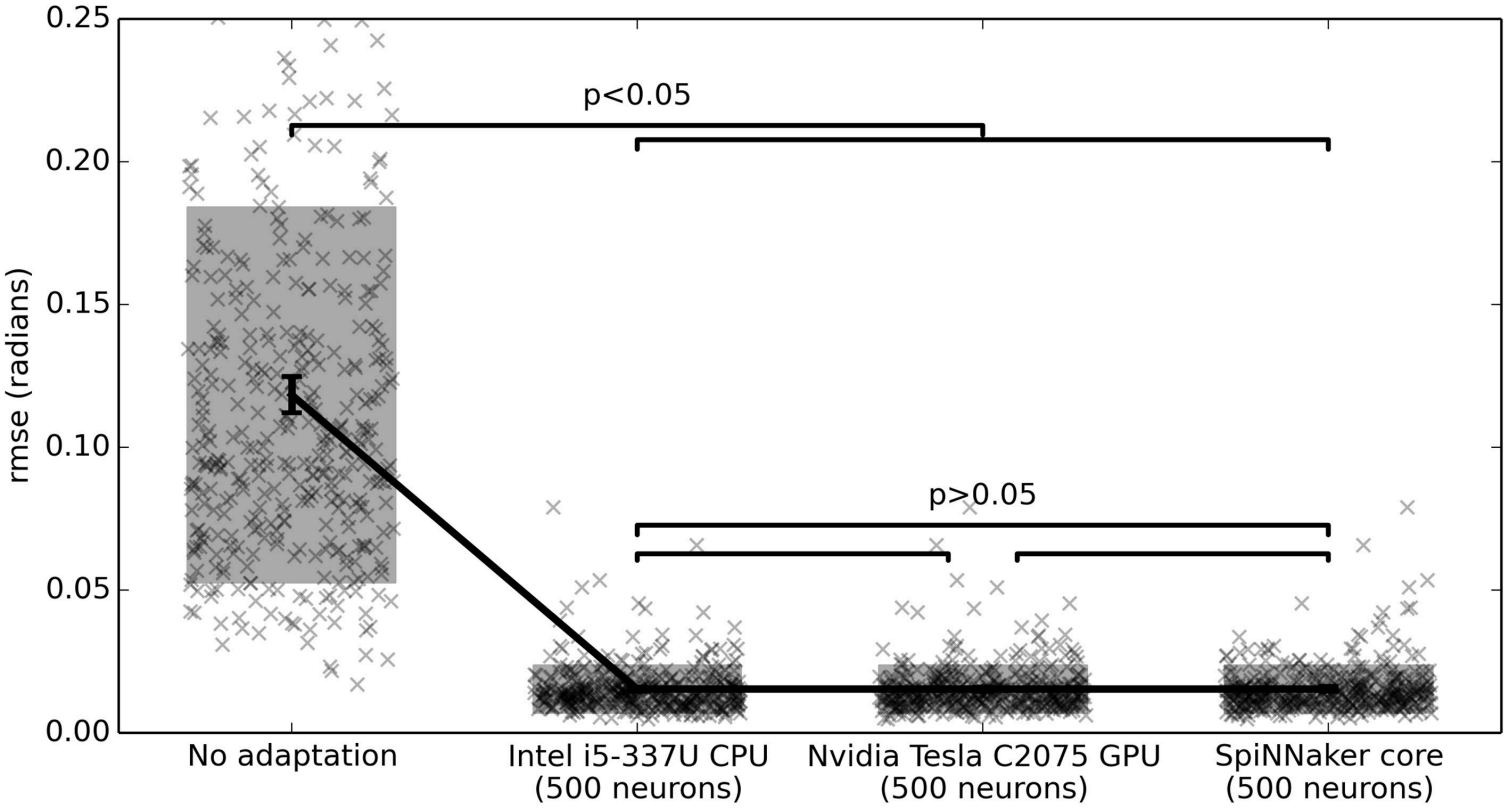
**Benchmark results comparing three hardware systems**. Each system is running 500 neurons. The hardware do not statistically significantly differ, but are all statistically significant improvements over no adaptation (*p* < 0.05; two-tailed *t*-test with Bonferroni correction; 400 samples per condition). Scatterplots show individual runs (with random jitter on the x-axis to avoid overlap), the shaded area is the mean plus or minus the standard deviation, and the 95% confidence interval of the mean is shown.

For example, Figure 5 shows the root-mean-squared error (rmse) between *q* and *q*_*d*_ for three different hardware systems. For this benchmark, *t*_*q*_, *t*_*u*_, τ_*q*_, and τ_*u*_ are chosen from *U*(0, 0.01) (the uniform distribution), σ_*q*_ and σ_*u*_ are from *U*(0, 0.1), and β, γ, η, and ζ are all *N*(0, 1). As discussed above, *K*_*f*_ is 1 and *T* is 10. *q*_*d*_ is set to be Gaussian white noise with a maximum frequency of 1 Hz and RMS power of 1. Each simulation is run for 20 s, and the error is computed on the last 10 s.

For each of three hardware platforms, we implemented the neural control system with the learning rule described above. That is, we started with a standard non-neural PD controller that produced an output *u*. The state information *q* was fed into a group of neurons using randomly generated input weights, producing output activity *A*. The actual output to the motor was *u* + *Ad* where *d* is a vector of learned weights, initialized to all zeros. The learning rule was Δ*d* = α*A* × *u*, and the learning rate α was fixed at 0.001.

The first hardware tested on this benchmark is an Intel i5-3337U CPU running at 1.80 GHz. This is not neuromorphic hardware, but provides a useful baseline. The learning algorithm was implemented using Nengo, a software toolkit for developing large-scale neural models that can be run on various hardware platforms (Bekolay et al., 2014). For the neuron model, we used 500 spiking Leaky-Integrate-and-Fire (LIF) neurons.

The second hardware used to generate Figure 5 is an Nvidia Tesla C2075 GPU, hardware that is often used for special purpose computing and neural network simulations. The same Nengo implementation was used, but retargetted to run on the GPU using OpenCL, with the same neuron model and number of neurons as the CPU.

The third hardware system benchmarked is SpiNNaker (Furber et al., 2014). This neuromorphic hardware consists of 18 ARM processors on a single chip, optimized for running neural models. Thanks to a SpiNNaker implementation for Nengo (Mundy et al., 2015), the same Nengo implementation that was used on the CPU and GPU is run on this hardware as well. Importantly, while the basic neuron model is the same, the actual implementation of this neuron model on SpiNNaker is very different from the implementation on the CPU and GPU, in that it relies on fixed-point computations and an asynchronous on-chip communication system.

All three hardware systems drastically improve performance on this task, as compared to the non-adaptive controller.

### 4.1. Computational power benchmark

**Figure 6:**
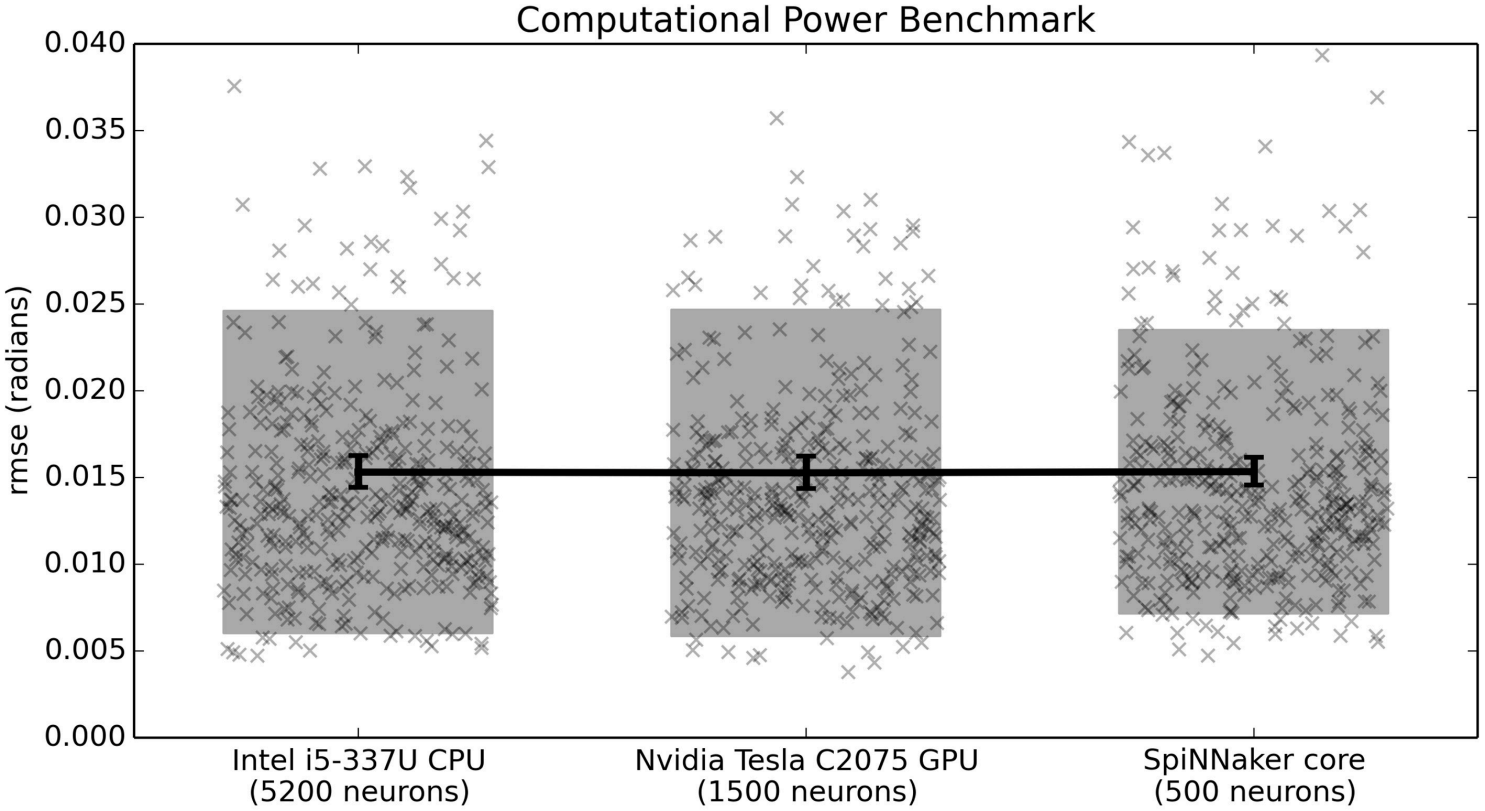
**Benchmark results comparing three hardware systems in terms of their performance when running as many neurons as they are capable of in real time**. Scatterplots show individual runs (with random jitter on the x-axis to avoid overlap), the shaded area is the mean plus or minus the standard deviation, and the 95% confidence interval of the mean is shown. In this case, there is no statistical difference between the three hardware systems (*p* > 0.05; two-tailed *t*-test with Bonferroni correction; 400 samples per condition).

In Figure 5, all three systems perform equally well. This means that the timing and accuracy differences between the fixed-point asynchronous SpiNNaker implementation and the floating-point synchronous CPU/GPU impementations do not affect performance on this task. However, on that benchmark all three systems are implementing exactly 500 neurons. This demonstrates that the differences in neuron model across that hardware does not significantly impact performance. Given that closed-loop models rely on real-time simulation, it is also important to determine how many neurons each piece of hardware is capable of running in real time. This is shown in Figure 6. With the current implementation, including both the Leaky Integrate-and-Fire neuron model and the learning rule, the CPU can run 5200 neurons, the GPU 1500 neurons, and a single SpiNNaker core can run 500 neurons (or 500 × 16 = 8000 neurons for the whole chip). These values were measured empirically with the current versions of the reference Nengo implementation, the Nengo OpenCL implementation, and the Nengo SpiNNaker implementation (as of August 10, 2015). All other parameters are as before.

### 4.2. Computational efficiency benchmark

While it is possible to run large neural models on standard CPUs and GPUs, one of the primary advantages of neuromorphic hardware is its power efficiency. For this reason, the third benchmark normalizes the number of neurons based on power consumption. With a power budget of 1 W per chip (with 16 used cores), we estimate 0.0625 W for the 500 neurons used here and round up to 0.1W to be conservative. Neither the CPU nor the GPU are designed to run on that little power. For this reason, on this benchmark we scale the number of neurons by the power consumption of the hardware. For this power consumption we measure the difference between the idle power consumption and the consumption when running the benchmark. For the Intel i5-3337U this was 34–11.5 W = 22.5 W and for the Nvidia Tesla C2075 GPU this was 74–70 W = 4 W. This value is much lower than the peak power consumption supported by the GPU (215 W), indicating that the current implementation does not make extensive use of the GPU for this task. Indeed, initial analysis indicates that the main bottleneck is communication between the GPU and the environment (i.e., the minimal simulation), and we feel it is appropriate that this benchmark captures that limitation of the current GPU implementation. The GPU could easily run many more neurons than this in real time, if those neurons were not connected to an environment. However, that would not be useful for a closed-loop task.

**Figure 7:**
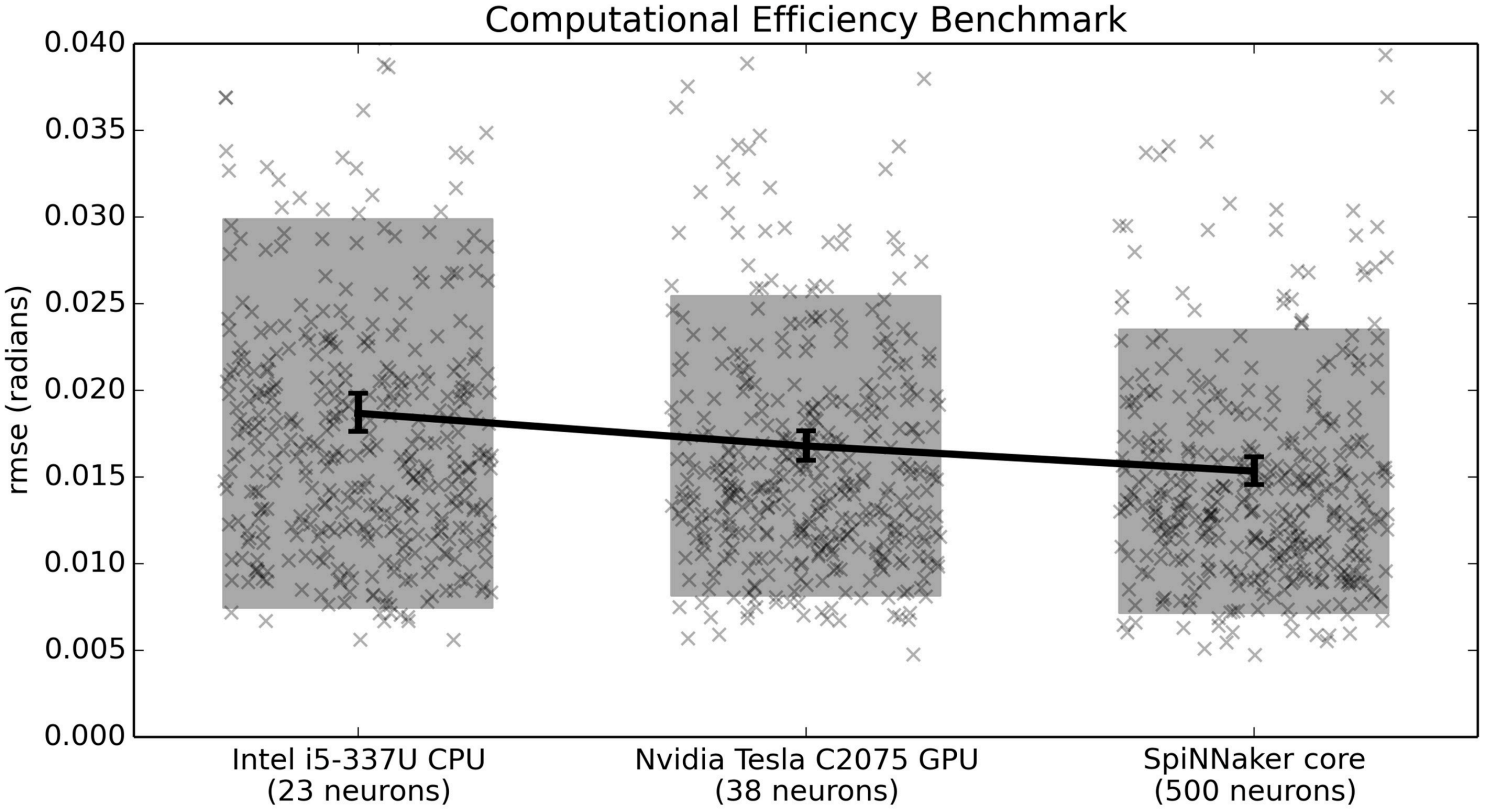
**Benchmark results comparing three hardware systems in terms of their performance when running as many neurons as they are capable of per 0.1 Watt of power consumption**. Scatterplots show individual runs (with random jitter on the x-axis to avoid overlap), the shaded area is the mean plus or minus the standard deviation, and the 95% confidence interval of the mean is shown. All differences are statistically significant (*p* < 0.05; two-tailed *t*-test with Bonferroni correction; 400 samples per condition).

Given the above considerations, the benchmark indicates that the CPU can run 23 neurons per 0.1 W, the GPU 38 neurons per 0.1 W, and SpiNNaker can run 500 neurons per 0.1 W. As shown in Figure 7, while the GPU outperforms the CPU on this task, the neuromorphic hardware outperforms both of them.

### 4.3. Communication delay benchmark

**Figure 8:**
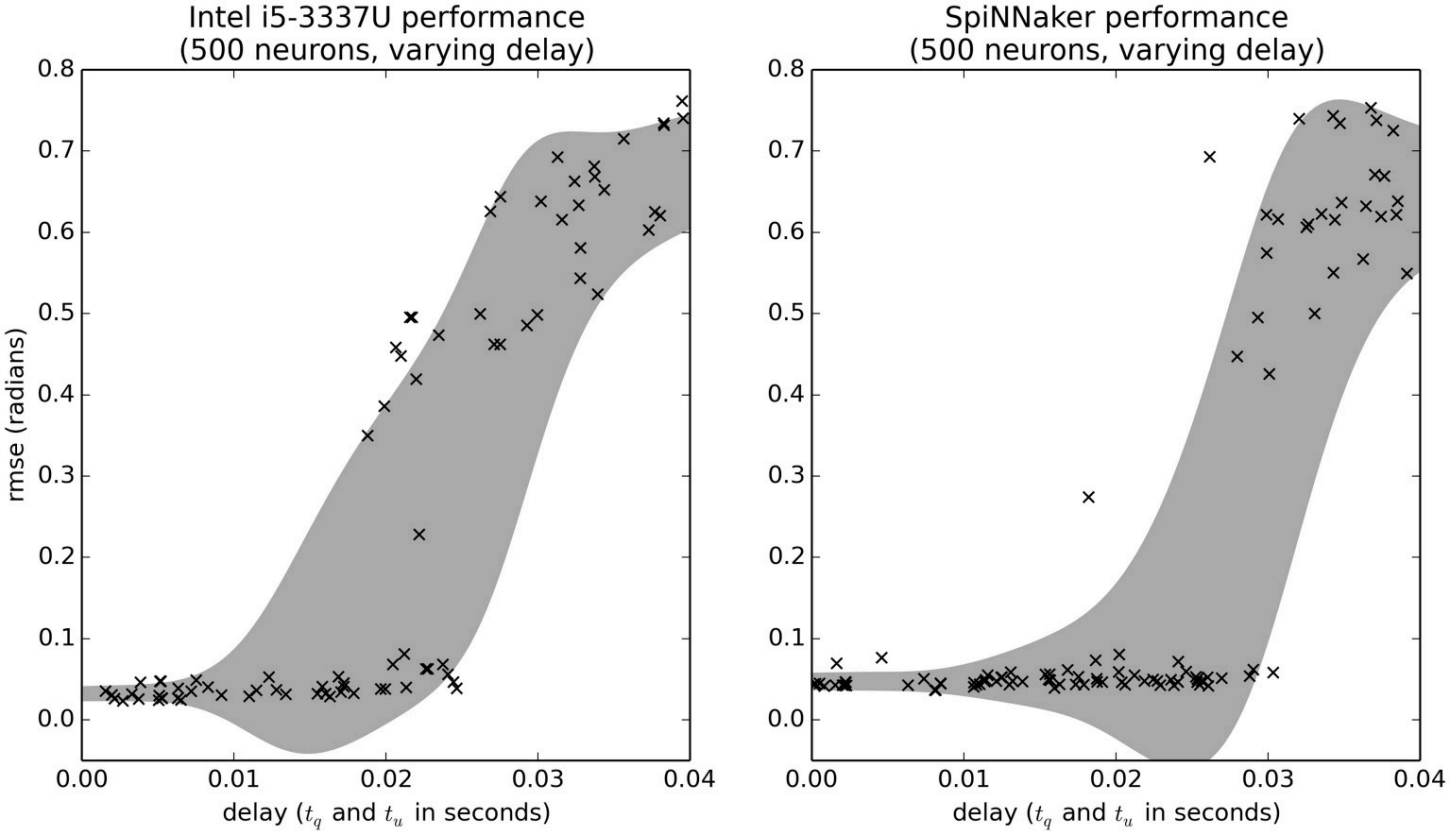
**Benchmark results comparing the effects of communication delay for the CPU and SpiNNaker systems**. The delays *t*_*q*_ and *t*_*u*_ are randomly varied. Shaded area is the mean plus or minus one standard deviation, smoothed with a Gaussian kernel of σ = 0.005.

We can also use minimal simulation benchmarks to examine the effects of various parameters in the model. For example, Figure 8 shows the effect of increasing the delays *t*_*q*_ and *t*_*u*_. Importantly, the range on the delay parameters is larger than in previous benchmarks [*U*(0, 0.04) rather than *U*(0, 0.01)]. Figure 8 shows that if the delay is short (less than 0.02), the controller performs well, and if it is very large (greater than 0.03), the controller performs poorly. However, for delays between 0.02 and 0.03, the controller sometimes performs well and sometimes performs poorly. The difference is due to the other random parameters in the system. Interestingly, SpiNNaker performs better on this task than the CPU (the GPU data is equivalent to the CPU and is not shown). This is somewhat surprising, as we are currently using the slow Ethernet interface to SpiNNaker, rather than the high-speed I/O system that is meant for motor control. Further analysis is needed to determine exactly why this is the case.

### 4.4. Scaling benchmark

As a final comparison, we look at how this algorithm scales as the number of neurons increases and as the number of controlled motors *N* increases. This is a crucial benchmark, as the complexity of the task itself quickly increases with *N* because the function computing the force applied at each joint is an interaction of *all* the joint angles *q*. Consequently, the number of parameter interactions in ithe external force that the neural system must learn to predict increases in exponentially as *N* increases. The quality of the control is thus dependent on how good an approximation the neurons can make of this complex non-linear function.

**Figure 9:**
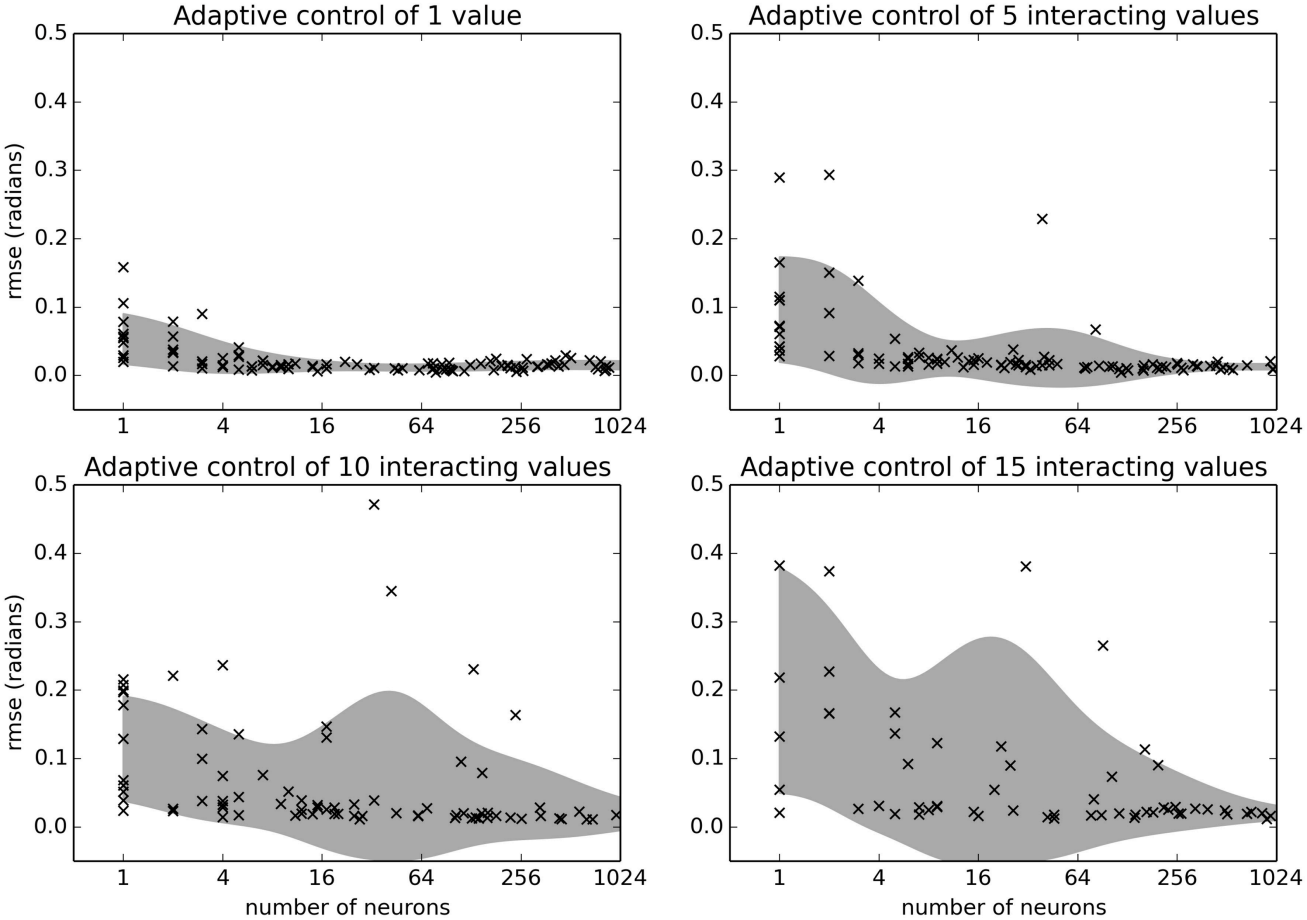
**Benchmark results examining the relationship between number of neurons and the number of simulated joints *N***. The benchmark was run on the Intel i5-3337U CPU. Shaded area is the mean plus or minus one standard deviation, smoothed with a Gaussian kernel of σ = 1 in the *log*_2_ domain.

As shown in Figure 9, adapting for unknown interacting forces on 15 joints is possible with 500 neurons. This gives an indication of how many neurons are needed for different tasks, and suggests that this controller could be used to control larger systems than those tested here.

## 5. Discussion

While the primary purpose of this paper is in describing the benchmarking methodology, it is also worth noting that these benchmarks indicate that the neuromorphic learning rule under investigation here is quite robust. As shown in Figure 9, even just 500 neurons can consistently adapt to control a *randomly generated* 15-joint body simulation, and deal with larger delays and noise than were seen in the example 1-joint physical embodiment. Since this learning system is robust across such a wide range of conditions, and since it is efficiently implementable in a wide variety of neuromorphic hardware, we feel it is worth further study. This must include both a wider variety of minimal simulation benchmarks and also a few more traditional benchmarks. These traditional benchmarks would be particular real physical systems (specific robot arms, for example), but testing on those would only reveal performance on those particular arms. As we argued here, benchmarking against a wide variety of randomly generated minimal simulation systems is needed to demonstrate the space of potential situations in which neuromorphic adaptive control performs well.

The benchmarks described above all use the same underlying minimal simulation as a way to characterize the overall performance of particular hardware across a range of situations. By adjusting the random distributions that define that range of situations, we generate different benchmarks that explore the capabilities of the systems in different ways. This allows for an explicit depiction of the sorts of conditions in which particular neuromorphic hardware performs well. After all, it is unlikely that one piece of neuromorphic hardware will be the best choice in all situations; rather, these benchmarks allow us to demonstrate the advantages and disadvantages of the hardware by looking at the same underlying system, but with multiple different distributions of parameters.

Python software for the minimal simulation and the full benchmarks are available at http://github.com/ctn-waterloo/ctn_benchmarks.

### 5.1. Benchmark improvements

The benchmarks presented here can be improved and further developed in several ways. Most obviously, we need to benchmark more hardware, and in particular we note that none of the systems tested here are analog neuromorphic hardware. While getting access to such hardware can be difficult, we believe the fact that our benchmark is easily shared with others as source code and interacts with existing hardware using a Python interface will help this process. Interestingly, it is worth noting that these benchmarks can also be run on software simulations of hardware (analog or digital), and could even be used to help form design decisions about hardware that has not yet been produced.

However, it is also clear that performance on these benchmarks is a result of a combination of the hardware itself, the algorithm being run, and the system that interfaces the hardware to the environment. Thus, for any given hardware, we can explore improvements to the algorithm (better choices for *e*, different learning rules, adaptive learning rates, adapting *K*_*p*_ and *K*_*d*_, etc.). For example, in the SpiNNaker hardware implementation not only can the neuron model be adjusted, but the distribution of the task across the multiple cores is also under programmer control. Furthermore, SpiNNaker provides a custom I/O interface for high-speed communication that could be used to reduce communication delay.

In addition, other classes of benchmarks could rely on expanded or completely different minimal simulations. For example, other physical systems could be used to calibrate the minimal simulation. This would lead to other classes of randomly generated external forces that may be more (or less) difficult for the neuromorphic system to learn. If we identify classes of tasks that we are likely to want to control, we can create modify those randomly generated forces to ones that are more appropriate for different tasks. For example, it may be of interest to randomly generate *N*-joint arms with random arm lengths and random masses, and derive (an approximation of) the actual forces that would be seen in those situations. In particular, we feel benchmarks based on the biologically-inspired “soft-robotics” systems (e.g., Pfeifer et al., 2013) would be particularly appropriate for neural control, given the complexity involved in generating traditional controllers for them.

### 5.2. Other benchmarks

While the particular minimal simulation shown here suggests that this adaptive control algorithm is worth further investigation, the overall goal of this paper is to present the general idea of using minimal simulation as a way to benchmark neuromorphic hardware. That is, we believe this same approach could be scaled up to other, more complex, closed-loop tasks. Importantly, benchmarking these other tasks would require both the creation of new minimal simulations *and* the specification of new algorithms suitable for performing those tasks. These algorithms would then be implemented with the neuromorphic hardware and connected to the minimal simulations to construct new benchmarks.

As a first step toward scaling up, consider the more complex task of controlling a system where the values to be controlled are not the joints themselves. For example, suppose we want to control the position of a hand *x*, but our output *u* only directly controls the joints *q* of an arm. The position of the hand *x* is some function of *q*, but this function may be unknown or highly complex. This is often expressed as $ẋ=J\left(q\right)\dot{q}$, where *J*(*q*) is the Jacobian. In order to successfully control *x* (the hand), the system needs to learn the relationship *J* that indicates how adjusting various joints *q* will affect the position of the hand. Crucially, there is a learning rule similar to the one discussed above that can learn this mapping (Cheah et al., 2006), and we have had some success in using it for particular arm control tasks (DeWolf, 2014). So far we have only tested this algorithm in the context of one particular arm, but it was successful in learning this relationship, and thus learning to correctly move its hand given an unknown arm geometry. To establish that this is a generally useful task for neuromorphic hardware, we need to benchmark this rule against a large family of different arms (and other systems to be controlled). This can be done by generating minimal simulations very similar to the one presented here; the main difference is that there would also be a randomly generated Jacobian function *J*(*q*). It should also be noted that in this context, the dimensionality of *x* and the dimensionality of *q* are separate variables. It may be that some algorithms work well when *q* is much larger than *x*, while others work best when they are similar. Exploring this relationship is fairly straightforward with minimal simulation, and would be an important result to know when choosing neuromorphic hardware for a particular new situation.

Given this, we believe that the combination of minimal simulation and neuromorphic hardware is useful for adaptive control problems in general, whether the adaptation is in terms of an additive bias term to compensate for external forces such as gravity (as seen in the benchmarks presented in this paper) or if it is in terms of learning the Jacobian term relating the controlled variables *q* to the desired target space *x* (as in the adaptive Jacobian model discussed in the previous paragraph). This should allow systems to adapt to both unknown external forces and to unknown bodily geometries. However, it is less clear whether this approach will scale to more complex robotics tasks.

One more complex robotic task where this approach might be applicable is navigation and obstacle avoidance. Here, we would need both a more complex minimal simulation for the environment, and an explicit neuromorphic algorithm capable of performing this avoidance. The minimal simulation itself would need to include some sort of sensory modality (vision, range sensing, or both), and movement in a two-dimensional environment (probably wheeled movement, for simplicity). To run such a simulation in real-time, we would use many of the same optimizations and simplifications used in Jakobi's original work (Jakobi, 1997). These included making separate simulations for corridors and intersections (rather than generic simulations for any possible geometries), using noisy lookup tables (rather than detailed physics simulations), treating collisions as failures (rather than modeling them), and using shifting random dot patterns for visual stimuli (rather than high-fidelity image rendering). Given Jakobi's success at building high-speed simulations over 20 years ago, we believe real-time simulations of this type are feasible now.

However, having such a simulation is only half of what is required. We would also need a control algorithm suitable for such a situation. This is, itself, a topic of much research, and there is no clear best approach. We have been exploring the use of reinforcement learning in neural models (Stewart et al., 2012; Rasmussen and Eliasmith, 2014), and note that these make use of the same learning algorithm as described here, with additional neural components needed to implement action selection. In this case, the learning rule would adjust the system's estimate of which action is most appropriate given the current sensory state. We are currently investigating this approach further.

As a more speculative possibility, we also intend to apply this approach to tasks involving classical and operant conditioning. Conditioning effects are extremely common in living creatures, and are clearly evident when animals are exposed to novel environments. As such, it is natural to define benchmark tasks involving learning the associations between sensory events in the environment (akin to classical conditioning) and the associations between actions and desired sensory states (akin to operant conditioning). In this case, the minimal simulations would consist of a set of small, controlled rooms with controllable buttons and stimuli, matching the sort of “Skinner Box” environments used in experimental psychology. The minimal simulation will also require a basic simulated body, capable of movement, pushing buttons, and observing stimuli. The tasks would consist of pairing stimuli together and determining if the learning algorithm is able to respond correctly. For example, a model might have a built-in response where it will salivate when presented with food. If the sound of a bell is paired with the presentation of food, it should learn to salivate with presented with just the sound of a bell. Importantly, there are extensive results showing the rate at which such associations are learned and un-learned in various animals. Furthermore, we would test the ability to learn associations that are separated in time (delayed conditioning), and to recover associations that had been previously learned (spontaneous recovery). Interestingly, there already exist neuron-based classical conditioning learning rules that may be suitable for such implementation, given their similarity to the learning rule used in the adaptive control benchmark (Verschure et al., 2003).

## 6. Conclusions

We have described a new method for benchmarking neuromorphic hardware that addresses the problem of reliably benchmarking complex tasks that involve interaction with an environment. This method involves building a minimal simulation; a simulation that is extremely simple in terms of required computation, but that has a high degree of adjustable variability. By benchmarking across a space of possibilities, we can identify hardware that performs well across that space, and is thus likely to be useful in real-world situations. In order to identify which real-world situations are covered by a minimal simulation, we can tune the variability in the simulation to particular physical systems.

We demonstrated this approach by defining a minimal simulation and a task appropriate for adaptive motor control. We presented an algorithm that can use neuromorphic hardware to improve performance on this task over that of a standard non-adaptive controller. Importantly, by measuring performance while adjusting the distributions of parameters in the benchmark, it is possible to characterize different aspects of the hardware, identifying how different aspects of the task affect performance for different hardware. This was demonstrated by providing five different benchmarks, each based on the same minimal simulation, but setting parameters in different ways. We believe this sort of flexibility is important in a benchmark method, as it lets researchers be explicit about what their hardware is good at, while still using the same basic and shareable benchmark framework.

Finally, we note that the benchmarking results show that this learning rule can consistently improve control performance across a wide variety of randomly generated situations, and is suitable for implementation on a wide variety of neuromorphic hardware. Given this promising result, we will be further evaluating it on specific physical embodiments, and comparing it to more complex variants of PID control.

## Funding

NSERC Discovery (grant 261453), ONR (N000141310419), AFOSR (FA8655-13-1-3084), Mitacs Postdoctoral Fellowship, Canada Research Chairs, Canadian Foundation for Innovation, Ontario Innovation Trust.

### Conflict of interest statement

The authors declare that the research was conducted in the absence of any commercial or financial relationships that could be construed as a potential conflict of interest.

## Footnotes

^1^Of course, for more complex benchmark tasks we may need sensory and motor capabilities that are beyond that of a simple Lego robot.

^2^*e* could also be chosen so as to regularly span the space of possibilities, or could be learned using some back-propagation of error method. Here, for simplicity, we only consider the approach of randomly selecting *e*_*i*_ and *b*_*i*_.
